# Expression of *methyl farnesoate epoxidase* (*mfe*) and *juvenile hormone esterase* (**jhe**) genes and their relation to social organization in the stingless bee **Melipona interrupta** (Hymenoptera: Apidae)

**DOI:** 10.1590/1678-4685-GMB-2020-0367

**Published:** 2021-08-09

**Authors:** Diana Vieira Brito, Carlos Gustavo Nunes da Silva, Livia Cristina Neves Rêgo, Gislene Almeida Carvalho-Zilse

**Affiliations:** 1 Instituto Nacional de Pesquisas da Amazônia, Grupo de Pesquisas em Abelhas, Programa de Pós-Graduação em Genética, Conservação e Biologia Evolutiva, Manaus, AM, Brazil.; 2 Universidade Federal do Amazonas, Centro de Apoio Multidisciplinar, Manaus, AM, Brazil.

**Keywords:** Juvenile hormone, eusocial bees, division of labor, caste determination, age polyethism

## Abstract

Social organization in highly eusocial bees relies upon two important processes: caste differentiation in female larvae, and age polyethism in adult workers. Juvenile Hormone (JH) is a key regulator of both processes. Here we investigated the expression of two genes involved in JH metabolism - *mfe* (biosynthesis) and *jhe* (degradation) - in the context of social organization in the stingless bee *Melipona interrupta*. We found evidence that the expression of *mfe* and *jhe* genes is related to changes in JH levels during late larval development, where caste determination occurs. Also, both *mfe* and *jhe* were upregulated when workers engage in intranidal tasks, but only *jhe* expression was downregulated at the transition from nursing to foraging activities. This relation is different than expected, considering recent reports of lower JH levels in foragers than nurses in the closely related species *Melipona scutellaris*. Our findings suggest that highly eusocial bees have different mechanisms to regulate JH and, thus, to maintain their level of social organization.

## Introduction

Juvenile Hormone (JH) is an isoprenoid compound that plays a remarkable role in the development (including metamorphosis), reproduction and regulation of distinct morphologies and behaviors of insect species (Hartfelder, 2000; Truman and Riddiford, 2002). JH is synthetized in the *corpora allata* (CA), which are endocrine glands located in the posterior regions of the insect head, and transported through the hemolymph to its site of action in target cells (Tobe and Stay, 1985; Gilbert *et al*., 2000). The presence or absence of JH at precise time points during the life cycle of insects is essential for its function. JH levels are ultimately regulated by two biochemical processes: JH biosynthesis and JH degradation (Gilbert *et al*., 2000).

JH production has been considered the main mechanism for controlling JH titers (Gilbert *et al*., 2000). In CA, JH is synthetized via the mevalonate metabolic pathway, which involves 13 enzymatic steps. The final and critical step of JH biosynthesis is catalyzed by the enzyme Methyl Farnesoate Epoxidase (MFE) (Bellés *et al*., 2005). JH degradation, on the other hand, is catalyzed primarily by one of two enzymes: JH Esterase (JHE) or JH Epoxide Hydrolase (JHEH) (Kort and Granger, 1981; Gilbert *et al*., 2000). JHE is released from fat body to act in hemolymph and other tissues, and it is considered the primary pathway of JH inactivation, while JHEH activity is secondary and exclusively tissue-bound (Tobe and Stay, 1985). 

For its wide function in the life cycle of insects and responsiveness to environmental stimuli, JH acquired pleiotropic functions within species, such as wing polyphenism in aphids, horn length in beetles, flight activity in moths, and regulation of social organization in many Hymenoptera species (Hartfelder, 2000). In highly eusocial bees (honey bees and stingless bees), social organization within colonies is based mainly on two processes: caste differentiation, in which females are morphologically specialized into reproductive or non-reproductive tasks (related to brood care and colony maintenance), and age polyethism, by which adult workers perform different tasks during their lives according to their ages (Robinson and Vargo, 1997).

In the honey bee *Apis mellifera*, caste differentiation is triggered by nutritional signals. Queen-destined larvae are fed with royal jelly during their entire larval development, while worker-destined larvae stop being fed with royal jelly in the late larval stages (Hartfelder and Emlen, 2012). These distinct nutritional signals generate changes in JH patterns. When JH titers are above a certain threshold, this leads to queen development (Hartfelder and Emlen, 2012). Age polyethism in honey bees, in turn, is mainly characterized by young workers performing tasks within the hive, such as brood care and nest maintenance (nursing); while older workers perform activities outside the hive, such as food collection and nest defense (foraging) (Robinson and Vargo, 1997). In *A. mellifera,* low hemolymph JH titers are associated with nursing tasks, while high JH titers are associated with foraging activities (Rutz *et al*., 1976; Huang *et al*., 1991). 

In most stingless bees, caste determination is also triggered by differential feeding, but only by quantitative, not qualitative differences. Females that receive larger quantities of food develop into queens (Camargo *et al*., 1976). In *Melipona* bees, however, caste is not primarily determined by differences in food quantity or quality (Kerr, 1950; Camargo *et al*., 1976; Maciel-Silva and Kerr, 1991). A genetic model with two-loci/two-alleles is the most accepted hypothesis so far to explain caste determination (Jarau *et al*., 2010). According to it, females that are homozygous at one or two loci turn into workers, while females that are double heterozygous (and well fed), develop into queens (Kerr *et al*., 1966; Kerr *et al*., 1975). Regardless of the initial input, high JH titers also lead to the development of queens in stingless bee species (Campos, 1978; Bonetti, 1984). Age polyethism in stingless bees, on the other hand, has a very similar pattern to the one observed in honey bees, with the transition from nest activities to foraging as workers gets older (Kerr and Santos-Neto, 1956). The endocrine basis of age polyethism was investigated in adult workers of *Melipona scutellaris* (Cardoso-Júnior *et al*., 2017). However, in contrast to honey bees*,* JH titers were significantly higher in nurses than in foragers.

Considering that JH has a crucial role for caste development and behavioral division of labor in highly eusocial bees, regulation of JH levels should be critical for the control of their social organization. Studies about the expression of *mfe* and *jhe* genes were done mainly with honey bees, with evidence that the transcription of these genes might be key regulation points of JH titers in the social organization context (Mackert *et al*., 2008; Bomtorin *et al*., 2014). Thus, this study aimed to investigate the transcriptional regulation of *mfe* and *jhe* genes in critical moments for caste differentiation and age-related division of labor in adult workers, and its importance for social organization in the Amazonian stingless bee *Melipona interrupta*.

## Material and Methods

### Bee sampling

Larvae and adult samples of *M. interrupta* were obtained from the meliponary of the Bee Research Group (GPA) located at the National Institute of Amazonian Research (INPA), Manaus/AM/Brazil (-3.0973067, -59.9847846). Larvae were collected at the second and the third instar, which is the last (and longest) in *Melipona* bees (n = 30) (Dias *et al*., 2001). The subdivisions of the third instar were classified based on the food amount/consistency and the presence/absence of feces within the brood cells, in accordance with previous studies with *Melipona* bees (Amaral *et al*., 2010; Cardoso-Júnior *et al*., 2017) (Table 1). Adult workers were sampled at three stages (n = 27). Newly emerged workers were collected after they hatched from brood cells; nurses were captured while constructing or provisioning cells with larval food; and foragers were caught while returning to the nest with nectar or pollen. All individuals were stored at -80°C after sampling.

**Table 1:** ‒ Characterization of the late larval stages in *Melipona interrupta.* The stages were classified according to Amaral *et al*. (2010) and Cardoso-Júnior *et al*. (2017).

Stage	Characteristics
L2	2^nd^ instar larvae
L3.1	3^rd^ instar larvae in the beginning of the last feeding period (brood cells with liquid food)
L3.2	3^rd^ instar larvae in the middle of the last feeding period (brood cells with viscous food)
L3.3	3^rd^ instar larvae in the end of the last feeding period (brood cells with solid food)
LPD	3^rd^ instar pre-defecating larvae, in the beginning of the cocoon-spinning period (brood cells without food)
LD	3^rd^ instar defecating larvae, in the cocoon-spinning period (brood cells with feces)

### Identification of **mfe* and *jhe* transcripts in *M. interrupta**

Total RNA was extracted from whole body of each individual with the SV RNA Isolation System kit (Promega), following the manufacturer’s instructions. RNA samples were quantified in the spectrophotometer Nanodrop 2000 (Thermo Fisher Scientific) and their integrity was verified by electrophoresis in agarose gel 1% (w/v). First-strand cDNA synthesis was made from 1 µg of total RNA using the Improm-II™ Reverse Transcription System kit (Promega).

Since there was no genome sequence available for *M. interrupta,* primers were initially designed to amplify and sequence partially the cDNA of *mfe* and *jhe* in this species*,* based on the coding sequences of orthologous genes from the closely related species *M. quadrifasciata* (Table S1). The OrthoDB: Database of Orthologous Groups program was employed to search for the *mfe* sequence in the genome of *M. quadrifasciata,* using *mfe* sequence of *Apis mellifera* as query (GenBank accession number: NM_001327966.1). The *jhe* sequence of *M. quadrifasciata* was obtained through a search directly in GenBank (accession number: EU099312.1). Sequencing was carried out on an ABI 3130 Genetic Analyzer (Applied Biosystems) with Big Dye^®^ Terminator v3.1 Cycle Sequencing kit (Applied Biosystems), following manufacturer’s instructions. The obtained sequences were compared to the sequences deposited in GenBank database using the Blast tool.

### Expression of **mfe* and *jhe* in larvae and adults of *M. interrupta**

The expression of *mfe* and *jhe* was analyzed using the method of quantitative real time PCR (two-step qRT-PCR). Primer sequences for *mfe* and *jhe* amplification were specific for *M. interrupta* (Table S1). Amplification detection was made with the Platinum^®^ SYBR^®^ Green qPCR Super Mix-UDG kit (Invitrogen), using the following reagent concentrations: 0.8X of SYBR^®^ green master mix, 0.5 µM of ROX reference dye, 0.4 µM of each primer, cDNA (0.25 µL generated from 1 µg of total RNA) and nuclease-free water q.s. 12.5 µL. Each reaction was done three times (technical replicates). Reactions were carried out on a Step One™ Real-Time PCR System (Thermo Fisher Scientific) in the following conditions: 50 °C for 2 min, 95 °C for 10 min, and 40 cycles of 95 °C for 15 s and 60 °C of 30 s. Melting curves were made at the end of each run to confirm the specificity of PCR products. Amplification efficiency (E) was estimated by using standard curves from a 1:3 dilution series of a representative sample and applying the equation E = 10^[-1/slope]^. E values were 98% and 109,2% for *mfe* and *jhe* primers pairs, respectively.

Expression levels were measured by the relative quantification method. *rpL32* (ribosomal protein L32) and *act* (actin) were used as reference genes for normalization, which were already experimentally validated as genes with stable expression in all developmental stages in *M. interrupta* (Brito *et al*., 2015). Primers for *rpL32* (previously named *rp49*) and *act* amplification were taken from literature data (Lourenço *et al*., 2008; Brito *et al*., 2015). Relative gene expression was calculated using the formula proposed by Pfaffl (2001), which allows the adjustment of differences between amplification efficiencies of target and reference genes. A geometric mean was applied in that formula to use two reference genes instead of one (Vandesompele *et al*., 2002; Hellemans *et al*., 2007) (Figure S1). The arithmetic means of Cq values obtained from second instar samples (larval stage analysis) and newly emerged workers (adult stages analysis) were used as calibrator.

### Statistical analyses

Variation in expression levels of *mfe* and *jhe* among the distinct periods of late larval development (five biological replicates in each category; n = 30) was tested by the statistical model of Analysis of Variance (ANOVA). Moreover, a Linear Regression Analysis was made for both genes to test if the relation between expression levels and larval stages could fit into a line or curve. Polynomial terms were included when patterns were curvilinear. Larval periods were considered an ordinal variable in these analyses. The relation between *mfe* or *jhe* expression and age-related division of labor among workers (nine biological replicates in each category; n = 27) was tested using ANOVA, followed by Tukey HSD test to identify differences between each group pair. Relative expression ratios were transformed into logarithmic values prior to statistical analysis in order to increase residual homogeneity of variance and normality. The significance of statistical tests was considered at the 0.05 level. All statistical analyses were made with the software R version 3.6.3 (R Core Team, 2020).

## Results

First, we confirmed the identity of *mfe* and *jhe* transcripts amplified in *M. interrupta.* The *mfe* sequence shared a high identity level with the orthologous genes in other bees, especially in the closely related species *M. quadrifasciata* (97%) and *M. scutellaris* (97%), but also in the bumble bee *Bombus terrestris* (86%), and in *A. mellifera* (81%). The *jhe* sequence was almost identical to its ortholog in *M. quadrifasciata* (99%), and also shared a high percentage of identity with the ones in *M. scutellaris* (98%), in the stingless bee *Scaptotrigona depilis* (97%), and in *A. mellifera* (86%).

Then, we asked if the expression of two genes related to JH metabolism varies in relation to five physiological and behavioral subdivisions of late larval development in *M. interrupta* (Table 1): the penultimate instar (“L2”); the progressive feeding periods of the last larval instar (“L3.1”; “L3.2”; “L3.3”); and the two periods within cocoon-spinning stage (larvae pre-defecating “LPD”; defecating “LD”). *Melipona* bees, unlike honey bees, are not raised in brood cells of different sizes (Hartfelder *et al*., 2006), thus it was not possible to discriminate the sex and caste of individuals at the larval stages. 

**Figure 1:**
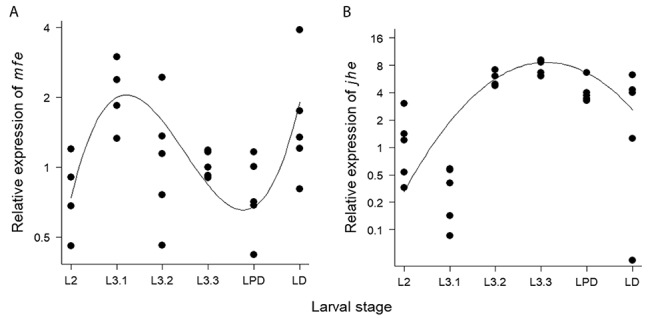
‒ Relative expression levels of *mfe* (**A**) and *jhe* (**B**) through late larval development in *Melipona interrupta*. Each point represents an individual (n = 30). The sex and caste of the individuals could not be identified at that stage. The x-axis shows the larval stages (see Table 1 for legends). The y-axis shows the relative expression ratios (i.e., fold changes) of target genes in experimental samples relative to calibrator samples. The mean of Cq (quantification cycle) values obtained from the second instar samples was used as calibrator. (**A**) Variation in *mfe* expression can be described by a cubic polynomial regression (Cubic Regression Model: r^2^ = 0.26, p = 0.01). (**B**) Variation in *jhe* expression can be described by a parabolic curve (Quadratic Regression Model: r^2^ = 0.25, p = 0.007).

We found that expression levels of both *mfe* and *jhe* varied significantly among the larval stages (ANOVA for *mfe*: F = 3.35, p = 0.02; ANOVA for *jhe*: F = 8.67, p = 8.3x10^-5^). Variation in *mfe* levels through late larval development could be best defined by a cubic polynomial regression (Cubic Regression Model: r^2^ = 0.26, p = 0.01). The *mfe* expression increased at the transition from the penultimate to the last instar (L2 and L3.1), decreased while larvae were progressively feeding (L3.2 and L3.3), and started to increase again at the defecating period of the cocoon spinning phase (LD) (Figure 1A). Variation in *jhe* expression could be best defined by a parabola (Quadratic Regression Model: r^2^ = 0.25, p = 0.007). In contrast to *mfe,* the *jhe* expression increased during the progressive feeding periods (L3.2 and L3.3), and gradually decreased during the cocoon-spinning phase (LD, especially) (Figure 1B).

Next, we investigated if *mfe* and *jhe* expression is related to age-related division of labor among workers. We analyzed the expression in three groups: individuals within the first hours after emergence (newly emerged); workers that were carrying out activities within the nest (nurses); and workers that were performing tasks outside the nest (foragers). 

**Figure 2:**
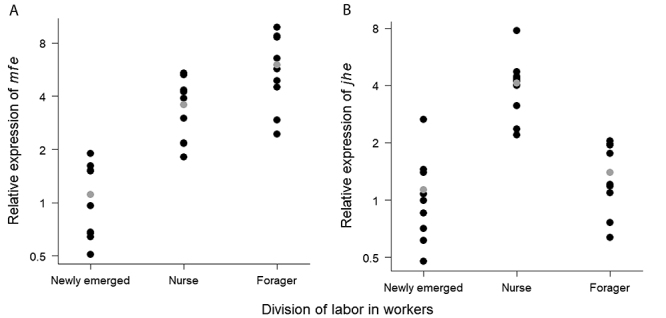
‒ Relative expression levels of *mfe* (**A**) and *jhe* (**B**) in relation to age-related division of labor among workers of *Melipona interrupta*. Each point represents an individual (n = 27). The x-axis shows the groups of workers performing distinct activities. The y-axis shows the relative expression ratios (i.e., fold changes) of target genes in experimental samples relative to calibrator samples. The mean of Cq (quantification cycle) values obtained from the newly emerged workers was used as calibrator. Mean values of relative expression ratios are shown in gray. Expression of both *mfe* and *jhe* varied significantly among worker groups (ANOVA for *mfe*: F = 31.81, p = 1.8x10^-7^; ANOVA for *jhe*: F = 23.3, p = 2.39x10^-6^). (**A**) *mfe* expression increased significantly in nurses (Tukey test: diff = 1.74, p = 3x10^-5^) and foragers (diff = 2.45, p = 2x10^-7^) compared to newly emerged workers. However, there was no significant difference in *mfe* expression between nurse and forager stages (diff = 0.71, p = 0.08). (**B**) *jhe* expression increased significantly from newly emerged to nurse stage (Tukey test: diff = 1.95, p = 3x10^-6^) and decreased significantly from nurse to forager stage (diff = 1.58, p = 7x10^-5^). There was no significant difference in *jhe* expression between newly emerged and forager stage (diff = 0.37, p = 0.44).

We found that expression levels of *mfe* were altered when workers started to perform tasks (ANOVA: F = 31.81, p = 1.8x10^-7^). *mfe* expression was significantly upregulated in nurses (Tukey test: diff = 1.74, p = 3x10^-5^) and foragers (diff = 2.45, p = 2x10^-7^) in comparison to newly emerged workers (Figure 2A). However, there was no significant change in *mfe* expression at the transition from nursing to foraging activities (diff = 0.71, p = 0.08). Expression levels of *jhe*, in turn, showed major differences at the nurse stage (ANOVA: F = 23.3, p = 2.39x10^-6^). *jhe* expression was significantly upregulated from newly emerged to nurse stage (Tukey test: diff = 1.95, p = 3x10^-6^), and downregulated from nurse to forager stage (diff = 1.58, p = 7x10^-5^). No significant difference in *jhe* expression was found between newly-emerged and forager workers (diff = 0.37, p = 0.44) (Figure 2B).

## Discussion

### Expression of *mfe* and *jhe* suggests a role in modulation of JH levels during larval development of *M. interrupta*

Here we observed that the patterns of variation in gene expression throughout late larval instars were distinct between *mfe* and *jhe* genes in *M. interrupta*. The differences were more pronounced in the progressive feeding periods (L3.2 and L3.3) and pre-defecating larvae (LPD), when the relative expression of *mfe* was low and the relative expression of *jhe* was high. 

Recent data report fluctuations in JH titers obtained for larvae of *M. scutellaris* in the same stages listed here (L2 to LD) (Cardoso-Júnior *et al*., 2017)*.* It was observed that JH titers decrease at the transition to the last instar (L2 to L3.1), remain low during the subsequent stages (L3.2, L3.3 and LPD), and increase at the defecating period of the cocoon spinning phase (LD). The increase of *mfe* expression in LD observed in our study corresponds positively to the JH levels described for *M. scutellaris*. Also, the high levels of *jhe* expression observed in progressive feeding stages, as well as its decrease in LD stage, are inversely related to JH levels in *M. scutellaris*. Thus, the variation observed in the expression of both genes, especially of *jhe* at the transition to the defecating period of the cocoon-spinning phase, is compatible with the changes in JH titers observed in *M. scutellaris*.

Changes in JH (and also in ecdysone levels) at the transition to the last larval instar and the feeding period of holometabolous insects are critical for the determination of adult body size (Truman and Riddiford, 2002; Emlen and Allen, 2003). Also, endocrine changes at the cocoon-spinning (defecating period) and prepupal stage (not analyzed here) are crucial for determining the final size of adult organs and body parts. These are the periods where the structures that will form adult body parts, which are called imaginal discs, grow by cell proliferation (Emlen and Allen, 2003). Experiments with JH measurements in the honey bee *Apis mellifera* and in the stingless bee *Scaptotrigona postica depilis* have demonstrated that these two periods are critical for the differentiation between queens and workers, in which JH titers are several folds higher in queen destined larvae than in workers (Rembold, 1987; Rachinsky *et al*., 1990; Hartfelder and Rembold, 1991). 

In honey bees, it has been established that the critical window for caste-specific JH action is the transition from fourth to fifth instar, which is the last and longest instar in these bees (Dietz *et al*., 1979). Changes in JH titers at this period overlap with the nutritional switch from royal jelly to worker jelly in worker larvae. Consequently, queen larvae grow much larger in overall body size than workers during the feeding period (Hartfelder and Emlen, 2012). In stingless bees (non*-Melipona* species), queen larvae receive two or three times more food than workers, which makes them have an extended feeding period and higher JH levels. As expected, these queens also have a larger body size than workers (Hartfelder *et al*., 2006). 

In *Melipona* bees, however, nutritional signals are not determinant for caste divergence. Actually, queens have a smaller body size before mating (Kerr, 1950; Hartfelder *et al*., 2006). The critical period for caste determination in *Melipona* is the cocoon-spinning period (Hartfelder and Emlen, 2012). It has been demonstrated that JH application at the beginning of the cocoon-spinning period is sufficient to induce the development of *Melipona* queens with the same morphological (head/thorax size, eye width and hind leg structure) and reproduction-related traits (ovaries morphology, spermatheca volume and tergal glands patterns) as the natural ones (Campos *et al*., 1975; Campos, 1978; Bonetti, 1984; Bonetti *et al*., 1994). The variation patterns observed for JH titers in *M. scutellaris* (Cardoso-Júnior *et al*., 2017) and *mfe* and *jhe* genes in *M. interrupta* here are in accordance with these data from earlier studies. Taken together, those findings support the hypothesis that *mfe* and *jhe* expression plays a role in JH titers modulation during a critical moment for caste determination in *M. interrupta*.

### Expression of *mfe* and *jhe* in *M. interrupta* adult workers indicates a complex role of JH in the social behavior of stingless bees

Here we found evidence that both *mfe and jhe* are upregulated in nurses, but only *jhe* is downregulated at the transition from within-hive to foraging tasks in workers of *M. interrupta*. This might be an evidence that JH titers are higher in foragers than in nurses in *M. interrupta*, which would be in accordance with what is known for honey bee workers. 

In honey bees, it has been demonstrated that JH acts together with the major egg yolk protein, Vitellogenin (Vg), to coordinate the transition from nest bees to foragers (Amdam and Omholt, 2002). Hemolymph levels of Vg are inversely correlated to the JH titers: Vg is lower in foragers than in nurses (Rutz *et al*., 1976; Fluri *et al*., 1982). In honey bee workers, Vg is not synthesized to be used for egg production, since they rarely lay eggs in the presence of a queen (Robinson and Vargo, 1997). Rather, it acts as an endocrine factor (Nelson *et al*., 2007). This regulatory role of Vg and its relation with JH in honey bee workers are opposite to the general norm in insects, which states that JH plays a key role in the reproductive physiology of females (i.e., gonadotropic hormone) by stimulating Vg synthesis (Hartfelder, 2000). Thus, it has been proposed that the reproductive regulatory network was co-opted for the control of age polyethism during the evolution of honey bees (Page *et al*., 2012).

In the stingless bees *M. scutellaris*, hemolymph levels of both JH and Vg are significantly lower in foragers than in nurse bees (Dallacqua *et al*., 2007; Cardoso-Júnior et al., 2017). Interestingly, egg production by nurse workers is common in many stingless bees. Workers can lay two types of eggs: trophic eggs, which are eaten by the queen; and reproductive eggs, which generate haploid males (Bourke, 1988). Thus, Cardoso-Júnior *et al.* (2017) hypothesized that JH does not play a role in age polyethism of stingless bees, but rather that it kept its ancestral gonadotropic function. These authors also pointed out that JH regulates worker reproduction in bumble bees (Bloch *et al*., 2000), which is the sister group of stingless bees in the phylogeny of corbiculate bees (Kapheim *et al*., 2015), and suggested that its gonadotropic function was retained in the Bombini/Meliponini clade.

Nevertheless, there are still many open issues regarding possible JH roles in worker behavior of stingless bees. First, stingless bees do not present significant differences from honey bees in their age-related division of labor (Kerr and Santos-Neto, 1956). Second, egg-laying by workers is a highly variable feature among species of stingless bee. There are workers that do not produce eggs in the presence of the queen; workers are capable of laying trophic, but not reproductive, and vice-versa; workers that lay reproductive eggs only occasionally; and workers that contribute to most of the male production (Tóth *et al*., 2004; Hartfelder *et al*., 2006). The physiological regulation of that process must be flexible also. Furthermore, JH titers were measured in only one species of stingless bee. As pointed out by Cardoso-Júnior et al. (2017), JH measurement is a time and labor consuming method that is carried out only in a few laboratories, which has been a caveat to expanding this research to other stingless bees.

Taken together, our results raise the possibility that JH might play a role in the age-related division of labor in workers of *M. interrupta* and that JH function could be variable among stingless bees’ species, or even that JH levels might have more complex patterns and play different functions in age-defined workers within the same species. Our data still need support from JH measurements though, since other regulatory checkpoints after gene transcription could alter JH levels. Still, giving the diversity of behaviors displayed by workers of stingless bees, JH role in adult workers of eusocial bees must be a complex issue and further studies are required. 

### Expression patterns of *mfe* and *jhe* are variable among highly eusocial bees

Furthermore, our results demonstrated that gene expression of both *mfe* and *jhe* is significantly variable among the stages of the last larval instar, especially at the cocoon-spinning phase, which is critical for caste determination in *M. interrupta*. In *A. mellifera*, it has been demonstrated that expression of *jhe*, but not of *mfe*, plays an important role in the control of JH titers at larval stages. Mackert *et al*. (2008) found that the variation in *jhe* expression is in accordance with the fluctuations in JH titers during the last larval instars of *A. mellifera*. Later on, Bomtorin *et al*. (2014) observed that *jhe* expression and JHE activity are significantly higher in workers than in queens at the transition from fourth to fifth instar, when caste-specific differences in JH titers are at a maximum in *A. mellifera*. These same authors noticed that neither *mfe* expression nor that of other JH biosynthesis genes is significantly different between workers and queens at that period. 

We also found that gene expression of *mfe* and *jhe* is variable among distinct behavioral stages of adult workers in *M. interrupta*. However, only *jhe* is regulated at the transition from within-hive to foraging tasks in workers. In *M. scutellaris*, Cardoso-Júnior *et al*. (2017) observed that *mfe* expression did not vary between nurses and foragers, which is in accordance with our results. In *A. mellifera*, however, the regulation patterns were opposite to our results. Bomtorin *et al*. (2014) found that the expression of *mfe* and five other JH pathway genes are upregulated in foragers, while *jhe* expression was not significantly different between nurses and foragers. Thus, transcriptional regulation of *jhe* and *mfe* in larval and adult stages is variable between honey bees and *Melipona* bees, which suggests that mechanisms for regulation JH titers within the context of social regulation might not be conserved among bees.

In summary, our study provides support for *mfe* and *jhe* expression as important regulation points of JH levels at moments of larval development that are critical for caste determination in *M. interrupta*. The caveat in the identification of workers and queens in larval stages needs to be overcome for a better understanding of these issues. Interestingly, we found that regulation of *jhe,* but not *mfe,* is related to the nurse-forager transition in workers of *M. interrupta.* Moreover, our findings also suggest that highly eusocial bees have different mechanisms for regulating JH levels. Further investigations are required to provide deeper insights into the regulatory gene network that has evolved in the stingless bees group.

## Supplementary material

The following online material is available for this article:

Table S1 - Primers used in PCR (gene identification) and qRT-PCR (gene expression) for the analysis of *mfe* and *jhe* in *Melipona interrupta*.

Figure S1 - Mathematical model used for calculation of normalized expression quantities (NRQ) with two reference genes in qRT-PCR.
