# Supplementary material for: Expression of methyl farnesoate epoxidase (mfe) and juvenile hormone esterase (jhe) genes and their relation to social organization in the stingless bee Melipona interrupta (Hymenoptera: Apidae)
Source: Genet Mol Biol. 2021 Aug 9;44(3):e20200367. doi: 10.1590/1678-4685-GMB-2020-0367 (PMC8361248; doi:10.1590/1678-4685-GMB-2020-0367)
Supplement: Table S1 - [file 1415-4757-GMB-44-3-e20200367-s1.pdf]

Genetics and Molecular Biology

https://doi.org/10.1590/1678-4685-GMB-2020-0367

Supplementary Material to "Expression of *methyl farnesoate epoxidase* (*mfe*) and *juvenile hormone esterase* (*jhe*) genes and their relation to social organization in the stingless bee *Melipona interrupta* (Hymenoptera: Apidae)"

Table S1 - Primers used in PCR (gene identification) and qRT-PCR (gene expression) for the analysis of *mfe* and *jhe* in *Melipona interrupta*.

Primer	Method	Sequence	Annealing temperature	Amplicon size
MFE_Fw	PCR	5' TCTTTGGTGTATGTTCGCTGG 3'	59°C	497 bp
MFE_Rv		5' CGACATTATCCAGTTCTTCC 3'		
JHE_Fw	PCR	5' GTGCYAARTATCTCATGGATMG 3'	56°C	418 bp
JHE_Rv		5' TAYCTGAGACAACGYAYCATYTC 3'		
MiMFE_Fw	qRT-PCR	5' CCGAGAATCAGCCACAGG 3'	60°C	118 bp
MiMFE_Rv		5' AAGAAGAGGTCCAGGCACAG 3'		
MiJHE_Fw	qRT-PCR	5' TGGTTCGGTGGTGATCCAAA 3'	60°C	129 bp
MiJHE_Rv		5' TGTGCCGCTGATCGAGATAC 3'		
