# Supplementary material for: Expression of methyl farnesoate epoxidase (mfe) and juvenile hormone esterase (jhe) genes and their relation to social organization in the stingless bee Melipona interrupta (Hymenoptera: Apidae)
Source: Genet Mol Biol. 2021 Aug 9;44(3):e20200367. doi: 10.1590/1678-4685-GMB-2020-0367 (PMC8361248; doi:10.1590/1678-4685-GMB-2020-0367)
Supplement: Figure S1 - [file 1415-4757-GMB-44-3-e20200367-s2.pdf]

Genetics and Molecular Biology

https://doi.org/10.1590/1678-4685-GMB-2020-0367

Supplementary Material to "Expression of *methyl farnesoate epoxidase* (*mfe*) and *juvenile hormone esterase* (*jhe*) genes and their relation to social organization in the stingless bee *Melipona interrupta* (Hymenoptera: Apidae)"

$$NRQ = \frac{\mathrm{E_{GOI}}^{\Delta \mathrm{C_{q,GOI\,(mean\ calibrator\ -sample)}}}}{\sqrt{\mathrm{E_{REF1}}^{\Delta \mathrm{C_{q,REF1\,(mean\ calibrator\ -sample)}}} \times \mathrm{E_{REF2}}^{\Delta \mathrm{C_{q,REF2\,(mean\ calibrator\ -sample)}}}}}$$

Figure S1 - Mathematical model used for calculation of normalized expression quantities (NRQ) with two reference genes in qRT-PCR. E_{GOI} is the amplification efficiency of the gene of interest (*mfe* or *jhe*). E_{REF} is the amplification efficiency of reference genes number 1 or 2 (*act*/*rpL32*). $\Delta Cq_{,\,GOI}$ is the difference between Cq values of calibrator and experimental sample for the gene of interest. $\Delta Cq_{,\,REF}$ is the difference between Cq values of calibrator and experimental sample for the reference gene number 1 or 2. Here we used the arithmetic mean of a group of calibrator samples, which were chosen according to the condition being tested. An exponential function was calculated separately for GOI and REF genes, using efficiency value as the base and delta Cq as the exponent. Comparison between GOI and REF genes is expressed by a ratio in this model. To use two REF genes, a geometric mean of the exponential function of both REF genes was applied into Pfaffl model (28), as proposed by Hellemans *et al.* (29) and Vandesompele *et al.* (30).
